# Diet may influence the oral microbiome composition in cats

**DOI:** 10.1186/s40168-016-0169-y

**Published:** 2016-06-09

**Authors:** Christina J. Adler, Richard Malik, Gina V. Browne, Jacqueline M. Norris

**Affiliations:** Institute of Dental Research, Faculty of Dentistry, The Westmead Millennium Institute for Medical Research, University of Sydney, Westmead, NSW 2145 Australia; Westmead Centre for Oral Health, Westmead, NSW 2145 Australia; Centre for Veterinary Education, University of Sydney, B22 Regimental Drive, Camperdown, 2006 NSW Australia; Faculty of Veterinary Science, University of Sydney, McMaster Building B14, Camperdown, 2006 NSW Australia

**Keywords:** Oral microbiome, Feline, Diet, Next-generation sequencing, 16S rRNA

## Abstract

**Background:**

Periodontal disease is highly prevalent amongst domestic cats, causing pain, gingival bleeding, reduced food intake, loss of teeth and possibly impacts on overall systemic health. Diet has been suggested to play a role in the development of periodontal disease in cats. There is a complete lack of information about how diet (composition and texture) affects the feline oral microbiome, the composition of which may influence oral health and the development of periodontal disease. We undertook a pilot study to assess if lifelong feeding of dry extruded kibble or wet (canned and/or fresh meat combinations) diets to cats (*n* = 10) with variable oral health affected the microbiome.

**Results:**

Oral microbiome composition was assessed by amplifying the V1-V3 region of the 16S gene from supragingival dental plaque DNA extracts. These amplicons were sequenced using Illumina technology. This deep sequencing revealed the feline oral microbiome to be diverse, containing 411 bacterial species from 14 phyla. We found that diet had a significant influence on the overall diversity and abundance of specific bacteria in the oral environment. Cats fed a dry diet exclusively had higher bacterial diversity in their oral microbiome than wet-food diet cats (*p* < 0.001). Amongst this higher diversity, cats on dry-food diets had a higher abundance of *Porphyromonas* spp. (*p* < 0.01) and *Treponema* spp. (*p* < 0.01).

**Conclusions:**

While we observed differences in the oral microbiome between cats on the two diets assessed, the relationship between these differences and gingival health was unclear. Our preliminary results indicate that further analysis of the influence of dietary constituents and texture on the feline oral microbiome is required to reveal the relationship between diet, the oral microbiome and gingival health in cats.

**Electronic supplementary material:**

The online version of this article (doi:10.1186/s40168-016-0169-y) contains supplementary material, which is available to authorized users.

## Background

The microbiome of the gingival cleft is of great interest in human dentistry because the two most important diseases of the teeth and periodontium in human patients, dental caries and periodontitis [1], are related to changes in the relative contribution of various potentially pathogenic bacteria in the complex biofilm referred to as dental plaque [2]. As a result, there is considerable information on the oral microbiome of human patients, how this is associated with different disease conditions, and how this is influenced by diet [3, 4]. This includes studies using ancient deoxyribonucleic acid (DNA) which have shown that systematic changes in the microbiome are correlated with changes in diet, in both a contemporary and an evolutionary sense [5].

Cats, like humans, are very commonly affected by periodontal disease, with a consensus that it is the most common disease of feline patients in developed nations [6]. While periodontal disease is seen in cats of all ages, it is generally considered to progress with age, although its extent and severity are impacted on by such factors as diet and co-morbid disease (especially kidney disease and infection with feline immunodeficiency virus and/or feline calicivirus). Indeed, some feline diets are specifically formulated to prevent and/or ameliorate the severity of feline periodontal disease. Although cats do not get dental caries, they are commonly afflicted by resorptive lesions (RLs), the origins of which are poorly understood but are characterised by erosion of enamel, dentin and/or cementum. Feline skulls analysed in retrospective studies of museum and zoo specimens demonstrate a low prevalence of RLs before the 1960s, which may suggest causal relationships with altered husbandry of domesticated cats including feeding practices [7].

The third disease condition of the feline oral cavity is referred to as feline chronic gingivostomatitis (FCGS). Although there is strong evidence to support the involvement of feline calicivirus (FCV) in some cases, the inability to recreate the disease in a naïve population and the success of treatments such as full-mouth dental extractions in many cases have cast doubts on a singular role for FCV and raised suggestions that this disease may be influenced by the nature of the host’s response and derangements (dysbiosis) of the oral microbiological flora.

The microbiome of the gingival cleft impacts additionally on common and important feline disease conditions outside the oral cavity. Infections resulting from cat bites, in both feline and human patients [8], are typically polymicrobial with a preponderance of obligate anaerobes and facultative anaerobic bacteria, of which only some are cultivatable using routine laboratory methods. Likewise, infections of the upper and lower respiratory tract and pleura of cats often involve oropharyngeal flora, including facultative and obligate anaerobic bacteria. Thus, chronic sinonasal cavity disease, pneumonia and especially purulent pleurisy (pyothorax) can involve cultivatable and likely uncultivable anaerobic bacteria, as well as facultative anaerobic bacteria such as *Pasteurella* spp.

Early studies on cultivatable organisms within the feline oral cavity found shifts towards a higher proportion of anaerobic gram-negative rods in cats with higher gingival index scores [9, 10], with prominence of bacteria within *Bacteriodetes* such as *Porphyromonas* sp. possessing suitable virulence factors capable of causing periodontal disease, and with these virulence factors inciting an appropriate humoral immune response [11–13]. To date, there have only been a handful of in-depth genetic studies of the feline oral microbiome [14–16], and these have not considered the contribution of diet to the observed findings.

The present work evaluates the composition of the feline oral microbiome using next-generation sequencing (NGS) and, in a preliminary fashion, the contribution of diet to the composition of the microbiome. The two mutually exclusive dietary categories fed to cats for months to years were (i) dry extruded kibbles (highly refined, cereal-based, dehydrated rations) and (ii) wet (canned [any type] and/or fresh meat [uncooked chicken, or lamb or beef; off or on the bone] combinations, high in water content, protein and fat, but low in carbohydrate). Our findings are in agreement with recent published work on the composition of the feline oral microbiome [14–18] and show a tangible effect of diet on the overall diversity and relative frequency of occurrence of known periodontal pathogens.

## Results

We used in-depth genetic sequencing to analyse the composition of the oral microbiome in 10 owned cats. The phylogenetically informative 16S gene (hypervariable region V1-V3) was amplified from supragingival dental plaque samples from cats in the study cohort. Illumina sequencing of the 16S PCR products produced a total of 2,421,096 sequences with an average of 186,238 sequences per sample, post-quality filtering. These sequences had an average length of 487 base pairs.

### Feline oral microbiome composition

**Fig. 1:**
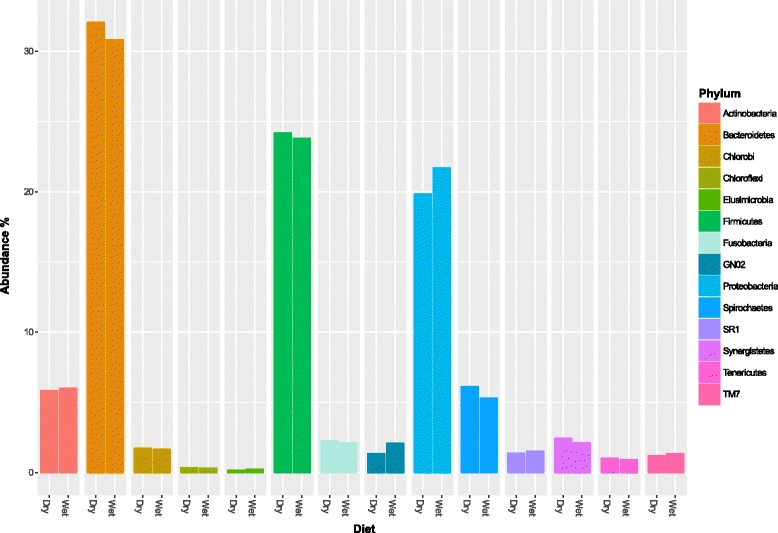
Relative abundance of phyla in the oral microbiome of cats on dry- and wet-food diets. The abundance of phyla was calculated from sequences classified as bacteria. These sequences were taxonomically assigned using the Greengenes database following operational taxonomic unit (OTU) classification with uclust (QIIME version 1.8.0). Relative abundances were calculated using normalised sequence data to account for varying sequence depth between samples (Phyloseq version 1.10.0)

**Fig. 2:**
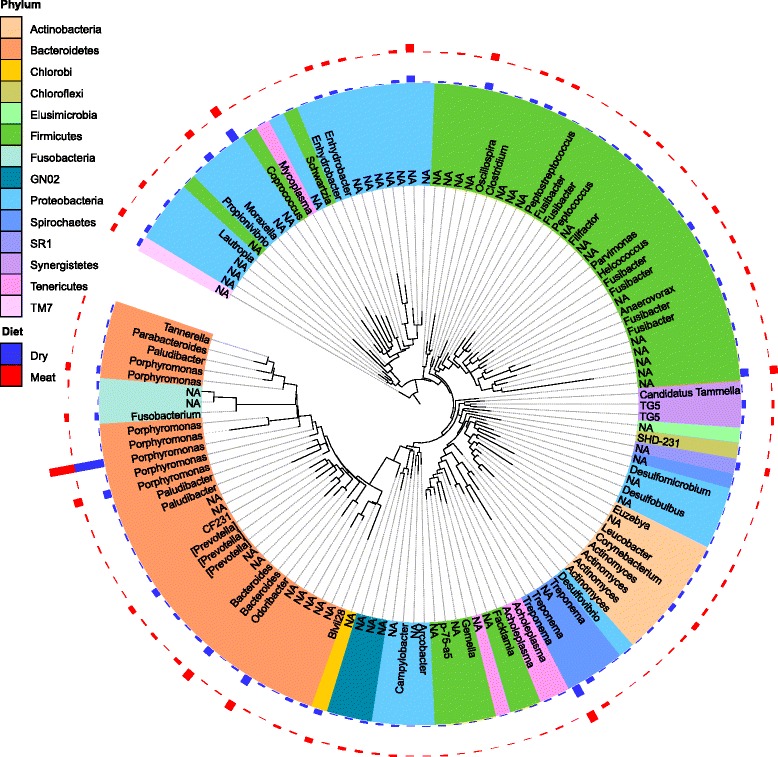
Phylogeny of sequences in the oral microbiome of cats on dry and wet diets. The phylogeny was generated using maximum likelihood (RAxML version 730) from representative sequences of the operational taxonomic units (OTUs) and graphically displayed in the Interactive Tree of Life (version 2.1). Sequences with less than 0.2 patristic distance between OTUs were collapsed (Phyloseq version 1.10.0). The abundance of genera in the dry- and wet-food diet groups displayed around the phylogeny was calculated from normalised OTU counts (Phyloseq version 1.10.0). Abbreviation: *NA* non-assigned

The cat oral microbiome contained a diverse array of bacteria from 14 phyla (Fig. 1). For each cat and diet group, the relative frequency of operational taxonomic units (OTUs) is presented in Additional file 1. Irrespective of diet, three phyla accounted for 76 % of sequences. These included *Bacteriodetes* (31 %), *Firmicutes* (24 %) and *Proteobacteria* (21 %). Amongst the phyla, we found a total of 411 bacterial OTUs across all the cats (Fig. 2). The majority (67 %) of these OTUs belonged to genera that occurred at abundances below 1 %. The remaining 33 % of OTUs had abundances above 1 % and accounted for the majority (76 %) of sequences. The most dominant OTUs across all the cats belonged to the *Porphyromonas* genus (14.9 %) followed by the *Treponema* (5.1 %) and *Fusibacter* (4.5 %) genera.

### Impact of diet on the feline oral microbiome

**Fig. 3:**
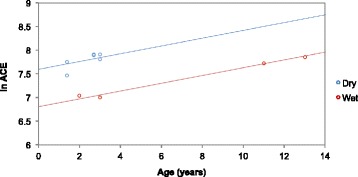
Analysis of covariance on the effect of diet type and age on the feline oral microbiome’s *α* diversity. Analysis of covariance (ANCOVA) was used to test the effect of dry and wet diets on *α* diversity, while controlling for age of the cats in R (version 3.1.2). Alpha diversity was calculated using the Abundance-based Coverage Estimator (ACE) metric, from operational taxonomic unit (OTU) abundance data (Phyloseq version 1.10.0). ANCOVA was performed on the log of ACE

The overall oral bacterial diversity was estimated for each cat (see Additional file 2). We used the alpha (*α*) diversity metric, Abundance Coverage Estimator (ACE), to assess species richness and the Simpsons Index to assess species evenness. We assessed whether the overall bacterial diversity differed between cats eating highly refined dry (higher carbohydrate content [both on a dry matter, total energy and absolute percentage basis], dehydrated, lower percentage of protein and fat on a percentage basis as fed) versus wet (lower carbohydrate content, high in water [approx. 70 %], variably higher in protein and fat) diets (see Additional file 3). Many of the dry extruded kibbles are sprayed with phosphoric acid to improve palatability, and such diets were the ones that tended to be fed to cats in the dry-food cohort. An analysis of covariance (ANCOVA) was conducted to determine if there was a statistically significant effect of diet on the cat’s oral microbiome ACE and Simpsons Index values, when controlling for age of the cats. We controlled for age because OTU diversity has previously been found to rise with increasing age [19]. We observed a significant effect of diet when controlling for age on species richness (ACE), *F*(2, 7) = 39.26, *p* = 0.0002 (Fig. 3), and evenness (Simpsons Index), *F*(2, 7) = 85.67, *p* = 0.00001. Cats on a dry-food compared to a wet-food diet had a higher diversity of OTUs in their oral microbiomes (ACE *p* = 0.0001, Simpsons *p* = 0.0001). Additionally, the overall bacterial diversity increased with increasing age (ACE *p* = 0.0002, Simpsons *p* = 0.0001).

**Fig. 4:**
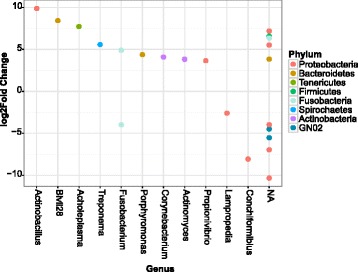
Differentially abundant genera between the oral microbiomes of cats on dry and wet diets. DESeq was used to test for the presence of differentially abundant operational taxonomic units (OTUs) in the oral microbiome of the cats on a dry- compared to wet-food diet (Phyloseq version 1.10.0). The DESeq test was applied to OTUs that had been filtered to remove singletons and species with an abundance below 0.005 %. The figure presents those OTUs that were found to be significantly different (*p* < 0.01) between the two diets. Positive log_2_-fold change values indicate enriched OTUs in the dry diet, and negative log_2_-fold change values indicate enriched OTUs in the cats eating a wet diet. Abbreviation: *NA* non-assigned

To determine what was causing the higher bacterial diversity in cats consuming dry food, we assessed the effect of diet on the abundance of specific bacteria. A differential abundance test, DESeq2 [20, 21], was used to compare the OTUs present in dental plaque samples from cats on dry-food to those on a wet-food diet. Of the 411 OTUs identified in the cat’s oral microbiomes, 23 were differentially abundant between the two diets (Fig. 4). Of these 23, 65 % of OTUs were significantly more abundant in plaque from cat’s consuming a dry diet. The most enriched bacteria in cats eating dry diets exclusively included OTUs from the following genera: *Actinobacillus* (*p* = 1.74 × 10^−8^), *Acholeplasma* (*p* = 0.0002), *Treponema* (*p* = 0.0087) and *Porphyromonas* (*p* = 0.0028). The remaining 35 % of OTUs were significantly more abundant in dental plaque from cats consuming a wet diet. These bacteria were primarily *Proteobacteria*, from the *Neisseriaceae* family (38 %), including *Conchiformibius kuhniae* (*p* = 0.0002). The differential abundance test indicated that diet alters the abundance of specific bacteria in the feline oral microbiome.

## Discussion

Our analysis of the oral microbiome is only the fourth NGS study in cats [14–16] and for the first time highlights the potential impact of diet on bacterial composition. Cats consuming dry kibble (dehydrated, highly refined, cereal-based, sprayed with oil and phosphoric acid) compared to a higher protein/lower carbohydrate wet diet (approx. 70 % water by weight) have a more diverse oral microbiome, with an enrichment of bacteria associated with both gingival health and periodontal disease. Our findings indicate that diets commonly fed to domestic cats in Australia [22] influence the feline oral microbiome make-up, although exactly how these composition changes relate to gingival health requires resolution.

Current understanding of the feline oral microbiome and what impacts its composition is limited by a lack of culture-independent studies. At a broad scale, we found a similar bacterial profile to published NGS [14–16] and Sanger sequencing [17, 18] studies of the feline oral microbiome, in terms of abundance of phyla and detection of genera. Our findings were most similar to Harris and colleagues’ study [14], which also found the dominant phyla to be *Firmicutes* (30 %), *Bacteriodetes* (22 %) and *Proteobacteria* (17 %). The present investigation and Harris et al.’s study [14] recovered a similar number of OTUs, 411 and 267, respectively, which was lower than the 10,177 OTUs recovered from NGS analysis of the feline oral microbiome by Sturgeon and colleagues [15]. This variation in bacterial diversity between studies is potentially attributable to the region of the 16S gene amplified. In both ours and Harris et al.’s study [14], the V1-V3 region of the 16S gene was sequenced, which is longer than the V4 region sequenced in Sturgeon and colleagues’ study [15]. At a fine scale, there was a variation in the abundance of OTUs recovered between the studies. For example, while we found *Porphyromonas* at an abundance of 15 %, Sturgeon et al. [15] found it at a frequency of 2 %, which may reflect variation in the gingival health between the cats assessed. The variation in the abundance of genera could also reflect differences between the studies in terms of OTU classification methods and reference databases used for species classification. Overall, while broad similarities in the feline oral microbiome were found between our and past NGS studies, the fine-scale differences highlight the extent of unknown information about what influences the composition of the cat oral microbial community.

We examined in a preliminary fashion one of the key factors thought to influence the composition of the human and feline oral microbiome, namely diet. While we detected differences in the abundance and diversity of bacteria between cats fed two broad categories of diets (dehydrated kibble versus well-hydrated meat-based or fresh meat rations), what these results mean for feline oral health is less clear. Cats consuming dry kibble exclusively, a popular choice in North America and to a lesser extent in Australia (13.4 %) [22], compared to a ‘wet diet’ were noteworthy in having a higher abundance of bacteria previously associated with both gingival health (including *Porphyromonas* and *Capnocytophaga* [14]) and periodontal disease (including *Porphyromonas* [11–13] and *Treponema* [14]), from analysis of the feline oral microbiome in different health states. Furthermore, we found that cats consuming a dry-food diet exclusively had higher bacterial diversity per se compared to cats fed canned wet food and/or fresh meat. Human oral microbiome studies have found higher bacterial diversity accompanying periodontal disease compared to oral health [23]. However, NGS analysis of the feline oral microbiome in the settings of gingival health and mild periodontal disease prior to our investigations found no difference in the overall diversity between these health states [14]. A critical deficiency in the literature is that cats with severe periodontal disease have not been subjected to this type of quantitative NGS analysis [14]. It is important to note that in this previous study [14], the age of the cats (ranging from 1 to 18 years) was not accounted for in the comparison of overall diversity between cats with gingival health and mild periodontal disease. We have shown that feline oral bacterial diversity significantly increases with advancing age of the patient, although the strength of this result is tempered by the small sample size of our study.

The lack of clarity from our study about the relationship between (i) the feline oral microbiome composition, (ii) the two disparate diets and (iii) gingival health probably relates to the limitations of our study, which includes the small sample size and lack of cats with a broad range of gingival health states. To adequately address the question of how diet impacts the feline oral microbiome and oral health, a case-matched study design would be ideal. This would involve comparing the oral microbiome of cats in two groups, one consuming a wet and one consuming a dry diet for identical time periods that were matched for age, breed and gingival health. A minimum of 50 cats per group would provide adequate power. A further consideration is that case recruitment would be challenging, as while periodontal disease is common, most people feed variable combinations of dry and wet diets, so finding cats where only one type of diet is consumed would require considerable time and effort.

Intuitively, the oral microbiome of any mammal, including humans, would be impacted by dietary composition. For example, increased carbohydrate intake has been associated with accelerated development of periodontal disease in controlled animal studies [24], ancient DNA analysis of fossilised human dental plaque from past agriculturist and hunter-gatherer populations [5] and human observational research [25]. All Felidae are obligate carnivores that have undergone reductive evolution through obtaining their dietary requirements from the flesh of prey. Prey are composed of protein, mineral and fat, the only carbohydrate being represented by plant ingesta in the gut and glycogen in the liver. Most animal tissues are generally well hydrated, with a water content of approximately 70 %. Changes in the oral microbiome would therefore be anticipated when diets were shifted by consumption of commercial cat foods (which were introduced in the 1970s and refined subsequently), especially dry diets based on carbohydrates from cereal (typically corn or wheat) mixed with rendered animal protein and coated with phosphoric acid, salt and fat to improve palatability. Cats fed commercial kibbles have a much higher intake of carbohydrate at 12 % [26] or higher, compared to cats consuming a prey-based diet, which has carbohydrate content in the order of 2 % [27]. In addition to food macronutrient composition, the mechanical properties of food are also likely to play a role, especially where diets contain meat on the bone, where there is a requirement to masticate, salivate (cat saliva is high in bicarbonate) and mechanically debride wearing dental surfaces due to the ‘flossing action’ of stripping meat from the bone. As well as physically removing plaque and even calculus, the debriding action of a flesh-eating lifestyle is likely to better buffer plaque pH [28]. The various points raised lend themselves to experimental interventions, specifically to determine the relative contribution of dietary composition as opposed to dietary texture.

## Conclusions

Commonly fed domestic diets influence feline oral microbiome composition, including the overall diversity and abundance of bacteria associated with both health and periodontal disease. From our preliminary analysis of the feline oral microbiome, we could not determine whether a dry kibble- or wet meat-based diet was preferable to ensure optimal gingival health. Findings from our pilot study indicate unequivocally that further research is warranted to determine the impact of dietary macronutrients, texture and related variables on the feline oral microbiome and to establish which diet type best promotes gingival health.

## Methods

### Population

We assessed the effect of diet on the oral microbiome of 10 cats. Supragingival swabs were collected as part of routine physical examination at the time of annual health checks. Consent was obtained from the owner of the cats, and a high standard (best practice) of veterinary care was adhered to. The characteristics of the cats, including age, breed, gender, diet, gingival health and household location, are provided in Table 1. The cats were fed either an exclusive dry extruded kibble diet (highly refined, cereal-based, dehydrated rations, generally a single brand only) or a composite wet diet (canned, sachet and/or fresh meat combinations; various commercial wet diets were fed, the variety representing variation within a brand and between different brands, and also a variable content of either fresh meat or fresh meat on the bone). Critically, cats in the ‘wet-food group’ were not ever fed kibbles. Dry-food diets consisted of 7.2–12 g/100 kcal of carbohydrate and 8.2–10 g/100 kcal of protein, while commercial wet-food diets consisted of 0.5–6.9 g/100 kcal carbohydrate and 7.1–15 g/100 kcal protein (Additional file 3). Stated another way, the percentage of carbohydrate as a percentage of what is fed was 0.6 to 4.8 % for commercial canned or sachet food, compared with 25 % for commercial premium kibbles. Cats fed a mixture of dry food and wet foods were deliberately avoided, although they would be a pertinent group in future work as this reflects the most common way owned cats are fed, at least in Australia [22].

**Table 1:** Characteristics of the participating cats

Sample ID	Age (years)	Breed	Gender^a^	Diet	Gingival score	Household
COM.02	11	BSH	Female	Wet	2+	Household 1
COM.04	3	DSH	Male	Wet	0	Household 2
COM.05	13	BSH	Male	Wet	2+	Household 1
COM.07	2	DSH	Female	Wet	1+	Household 2
COM.08	3	Ragdoll	Male	Dry	1+	Household 3
COM.09	3	Ragdoll	Male	Dry	1+	Household 3
COM.10	1.4	Burmese	Male	Dry	1+	Household 4
COM.11	1.4	DSH	Female	Dry	1+	Household 4
COM.12	2.7	DSH	Female	Dry	1+	Household 5
COM.13	2.7	DSH	Male	Dry	1+	Household 5

*BSH* British shorthaired, *DSH* domestic crossbred

^a^All cats were neutered

Gingival health status was scored on a 0–3 scale, with 0—normal gingiva with sharp, non-inflamed edges; 1—marginal gingivitis, minimal inflammation at free margin, no bleeding when pressure (from a cotton-tipped swab) was applied to the gingiva; 2—moderate gingivitis, wider inflammation at gingival margin, bleeding when pressure was applied to the gingiva; and 3—marked gingivitis, severe inflammation, bleeding present or absent when pressure was applied to the gingiva [16]. Examples of the gingival health states of a selection of the cats in our study are presented in Additional file 4. Cats were excluded from the study if they had undergone dental hygiene procedures in the preceding 12 months, were taking antimicrobials or immunosuppressants during the past 6 months, or had co-morbidities such as chronic kidney disease. One cat (COM.01), for which genetic analysis was performed, was excluded from statistical analysis. This cat had extremely low bacterial diversity and had several months prior to the study taken various courses of antimicrobials. This resulted in a total of 10 cats being included in statistical analyses.

### Dental plaque sampling

Supragingival plaque samples were obtained from each cat. Dental plaque was collected by inserting a sterile swab into the cat’s mouth and swabbing the gums (both upper and lower dental arcade) and teeth for 10–15 s. The swab was then immediately stored in a sterile 1.5-ml tube containing transport media and placed in a −20 °C freezer until DNA extraction was undertaken.

### Genetic analysis

Genetic analysis of the dental plaque samples (*n* = 10) included DNA extraction, amplification of the 16S gene and sequencing of these amplicons on the Illumina MiSeq platform.

*DNA extraction*: All biofilm samples were extracted using the PowerBiofilm™ DNA Isolation Kit (MoBio) according to the manufacturer’s instructions, with the addition of a 10-min incubation step at room temperature before the final centrifugation and elution to increase DNA recovery. All samples were co-extracted with blanks to monitor for contamination.

*Amplification of the 16S rRNA gene*: PCR was used to amplify the V1-V3 region (nucleotide position 27–519) of the 16S rRNA gene. The PCR conditions included 0.625 U of ThermoPol Taq (New England BioLabs) in a 25-μl volume using 10× ThermoPol Taq Buffer, 200 μM of each dNTP (Fermentas), 0.2 μM of each primer and 2 μl of DNA extract. The thermocycling conditions consisted of an initial enzyme activation step at 95 °C for 30 s, followed by 25 cycles of denaturation at 95 °C for 20 s, annealing at 54 °C for 15 s and elongation at 68 °C for 40 s, with a single final extension step at 68 °C for 5 min. Each set of PCRs included extraction and PCR blanks. All PCR products were visually examined by electrophoresis on 2.0 % agarose TBE gels. Positive PCR products were selected for Illumina sequencing.

*Illumina sequencing of the 16S amplicons*: Illumina sequencing was used to examine the microbial contents of the 10 dental plaque sample DNA extracts. Amplicons were sequenced on the Illumina MiSeq platform with 300 base pair, paired-end read chemistry.

### Sequence analysis

Sequence analysis of the Illumina data, including quality filtering, taxonomic classification and phylogeny generation, was undertaken in QIIME version 1.8.0 [29].

Quality filtering was used to remove sequences that contained ambiguous bases, had primer or barcode mismatches, contained homopolymers that exceeded six bases or had a minimum Phred quality score below 20. The quality-filtered sequences were checked for the presence of chimeras using usearch61 [30, 31] with Greengenes (version gg_13_8) [32] as the reference dataset. This was done to remove sequences containing errors produced during PCR.

Sequences which shared 97 % similarity were binned into OTUs using open-reference OTU picking via uclust [30], with Greengenes (version gg_13_8) as the reference dataset. Representative sequences from each OTU were taxonomically assigned using the RDP classifier and nomenclature [33]. Representative sequences were aligned using PyNAST [34] against the Greengenes reference dataset (version gg_13_8). This alignment was used to build a phylogeny with RAxML version 730 [35].

### Statistical analysis

Statistical analysis of the quality-filtered and classified sequence data was undertaken in R (version 3.1.2), primarily using the Phyloseq package (version 1.10.0) [36].

*Alpha diversity*: Within-sample diversity was estimated per sample on the quality-filtered data, which had not been submitted to any further pre-processing, such as removal of singletons. The *α*-diversity metrics, ACE and Simpsons Index, were calculated for all samples. To assess the impact of diet on *α* diversity, while controlling for age of the cat, we used an ANCOVA. This test assumes the ACE, and Simpsons Index values will be normally distributed. Residual plots of the ACE and Simpsons Index values indicated they were non-normally distributed. To improve the distribution, ACE and Simpsons Index values were log transformed. ANCOVA was performed on the log-transformed values of ACE and Simpsons Index in R.

*Pre-processing of sequences*: Before undertaking further statistical analyses, very low abundant sequences were removed in line with current recommendations [37]. We removed OTUs that were singletons and had an abundance below 0.005 % of all the sequences.

*Differential OTU test*: The DESeq2 package [20, 21] was used to test for the presence of differentially expressed OTUs between two diets. The test is a negative binomial generalised linear model (GLM), Wald statistic. The GLM is used to model the counts of OTUs per sample using a negative binomial distribution. The experimental design for the test was set to compare the OTUs from the dry- and wet-food diet groups. All OTUs that significantly (*α* = 0.01) differed in abundance between the two diets according to the DESeq test results were reported. All reported *p* values were adjusted for multiple comparisons using the Benjamini-Hochberg, false discovery rate procedure.

*Normalisation of OTU count data*: To account for variation in sequence depth between samples, we used a variance stabilised transformation (VST) to produce normalised sequence data by library size. All abundances of OTUs reported are from the VST data.

### Consent for publication

Not applicable.

### Availability of data and materials

Data will be deposited in the European Molecular Biology Laboratory (EMBL/EBI) Nucleotide Sequence Database on acceptance of the manuscript.

## Additional files

Additional file 1: Relative frequency (%) of OTUs per cat and per diet group. Abbreviations: non-assigned (NA) and standard deviation (SD). (XLSX 133 kb) Additional file 2: Alpha diversity metrics per cat. The alpha diversity metrics presented include the Abundance Coverage Estimator (ACE), which estimates species richness, and the Simpson’s Index that estimates species evenness. Abbreviations: Abundance Coverage Estimator (ACE) and standard error (SE). (XLSX 63 kb) Additional file 3: Nutritional data concerning commercially available wet and dry extruded rations available in Australia. The nutritional data provided include the metabolisable energy (ME), the percentage of carbohydrate in the food (% CHO), the percentage of carbohydrate on a dry matter basis (DM % CHO), and the amount of carbohydrate (CHO), fat and protein in the food in grams per 100 kcal. This information was collated meticulously by Dr. Linda Fleeman. Abbreviations: metabolisable energy (ME), carbohydrate (CHO) and dry matter (DM). (XLSX 14 kb) Additional file 4: Gingival health states of a selection of participating cats. (PDF 4685 kb)

## Abbreviations

DNA: deoxyribonucleic acid
FCGS: feline chronic gingivostomatitis
FCV: feline calicivirus
NA: non-assigned
NGS: next-generation sequencing
OTU: operational taxonomic unit
RLs: resorptive lesions
