# Supplementary figures and images for: Diet may influence the oral microbiome composition in cats
Source: Microbiome. 2016 Jun 9;4:23. doi: 10.1186/s40168-016-0169-y (PMC4899902; doi:10.1186/s40168-016-0169-y)

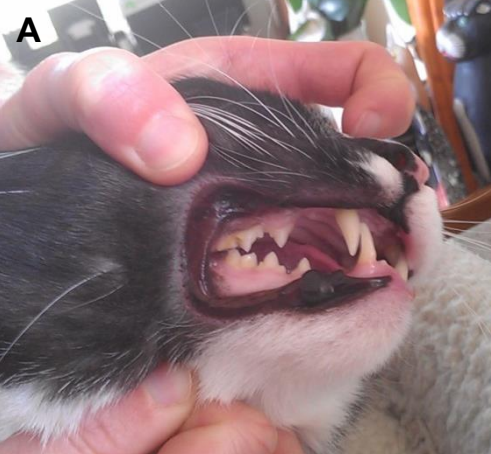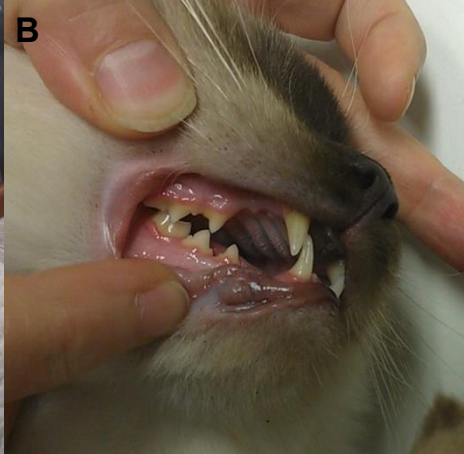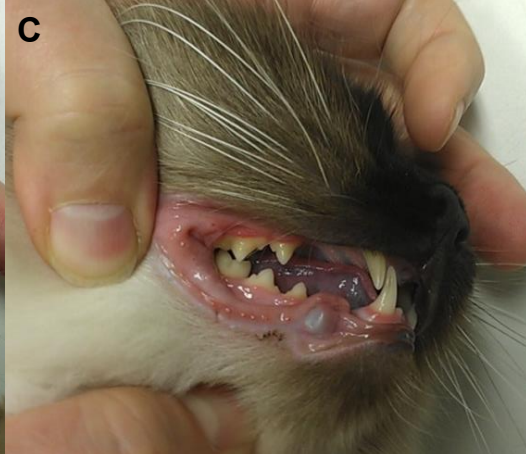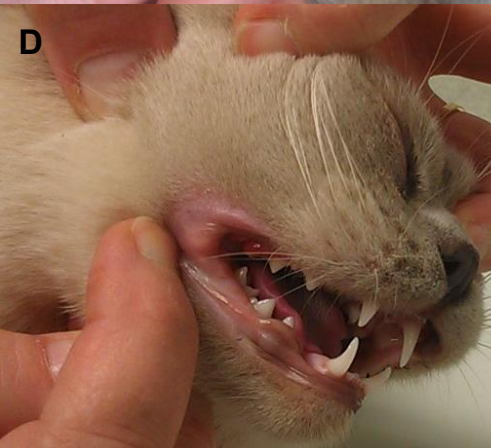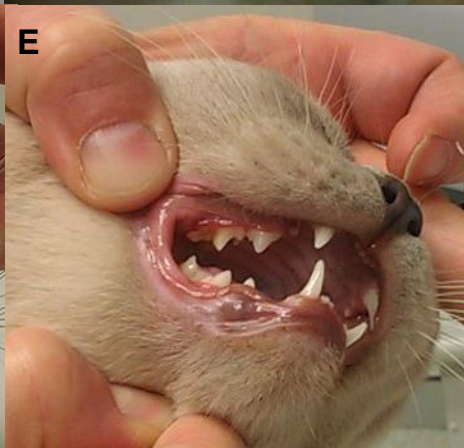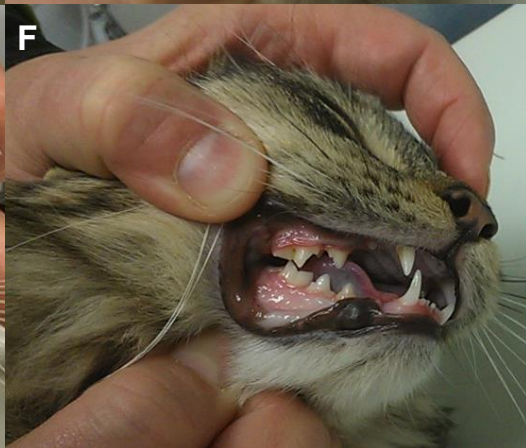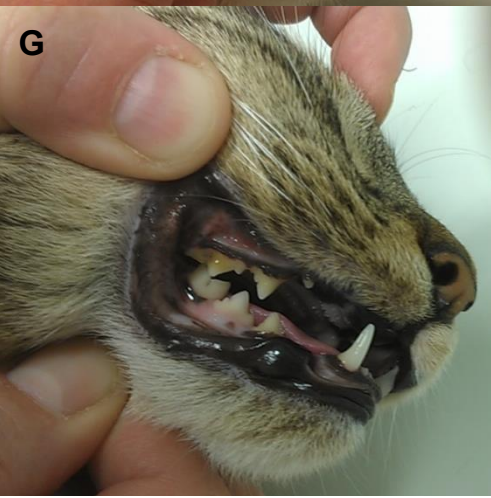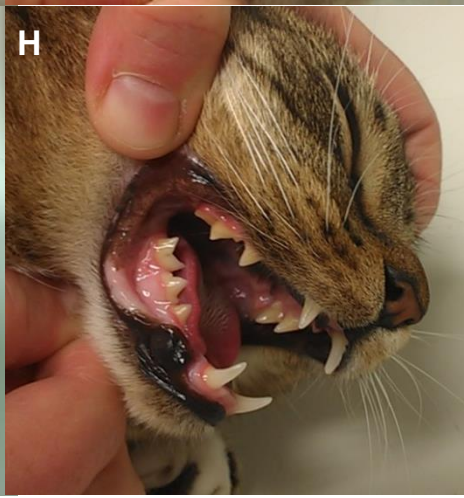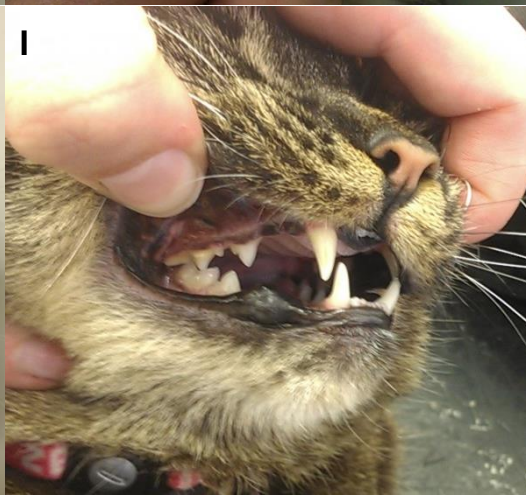

Supplement: Additional file 4: — Gingival health states of a selection of participating cats. (PDF 4685 kb) [file 40168_2016_169_MOESM4_ESM.pdf]
